# Lichen Sclerosus and Human Papillomavirus in Failed Hypospadias Repair: A Histopathological Case Series

**DOI:** 10.7759/cureus.106600

**Published:** 2026-04-07

**Authors:** Anabella Maiolo, Jimena Krikorian, Sebastian G Tobia González

**Affiliations:** 1 Urology, Sor Maria Ludovica Children's Hospital, La Plata, ARG; 2 Urology, Driscoll Children's Hospital, Corpus Christi, USA

**Keywords:** balanitis xerotica obliterans, hpv, hypospadias, lichen sclerosus, pediatric urology, urethral reconstruction

## Abstract

Background: Lichen sclerosus (LS) is a chronic inflammatory dermatosis that can affect the male genitalia and has been implicated in urethral and meatal complications. Its role in hypospadias surgery outcomes remains controversial. Additionally, a possible association between LS and human papillomavirus (HPV) infection has been suggested. This study aimed to evaluate the presence of LS, koilocytic changes, and HPV in tissue samples obtained during hypospadias surgery and to assess their relationship with postoperative complications.

Methods: A retrospective case series was conducted, including 11 pediatric patients who underwent hypospadias repair between 2015 and 2020 and had intraoperative biopsies demonstrating LS. A total of 13 tissue specimens were analyzed using histopathology and polymerase chain reaction (PCR) for HPV detection and genotyping. Postoperative outcomes were reviewed and correlated with the histopathological findings.

Results: The overall complication rate was 54.5% (6/11). LS was identified in all specimens. Koilocytic changes were observed in 76.9% of samples, and HPV DNA was detected in 46.1%, including serotypes 6, 16, 18, and 33. No significant association was found between the presence of HPV or koilocytic changes and postoperative complications (p>0.05).

Conclusions: LS was consistently identified in tissue samples obtained during hypospadias repair in this small series and may represent an underlying pathological factor in some patients. Although HPV was detected in a subset of cases, its clinical significance remains uncertain, as no association with postoperative complications was demonstrated. These findings should be interpreted with caution given the limited sample size. Further studies are needed to clarify the role of HPV and the impact of LS on reconstructive outcomes in hypospadias surgery.

## Introduction

Lichen sclerosus (LS), historically referred to as balanitis xerotica obliterans (BXO) when involving the male genitalia, is a chronic inflammatory dermatosis that affects the genital skin of both males and females. In males, LS may involve the foreskin, glans penis, and urethral meatus, and it has been associated with several urological conditions, including phimosis, meatal stenosis, and urethral strictures [1-3].

Histologically, LS is characterized by epidermal atrophy, dermal hyalinization, and inflammatory infiltration. In some cases, epithelial cells may show koilocytic changes, which have been interpreted as possible markers of human papillomavirus (HPV) infection [4]. The potential role of HPV in the pathogenesis of LS remains controversial, although viral DNA has been identified in a subset of lesions in both adults and children [5].

In pediatric urology, LS has gained increasing attention because of its potential impact on reconstructive surgery. Several studies have suggested that the use of affected penile skin during urethral reconstruction may increase the risk of postoperative complications such as meatal stenosis, urethral strictures, or fistula formation following hypospadias repair [6-8]. Despite advances in surgical techniques, hypospadias repair remains associated with a variable rate of postoperative complications, and underlying tissue characteristics and inflammatory conditions may influence surgical outcomes [9,10].

Despite growing interest in this topic, the available evidence remains limited and heterogeneous. In particular, there is a lack of studies that simultaneously evaluate the histopathological features of LS, the presence of koilocytic changes, and molecular detection of HPV in the context of hypospadias surgery. Furthermore, the clinical relevance of these findings in relation to postoperative complications remains unclear.

The aim of this study was to evaluate surgical outcomes in pediatric patients undergoing hypospadias repair in whom histopathological examination demonstrated LS. In addition, we investigated the presence of koilocytic changes and HPV in tissue samples obtained during surgery.

## Materials and methods

Between January 2015 and December 2020, a retrospective observational case series was conducted including pediatric patients who underwent surgical correction of hypospadias at a tertiary pediatric referral center. During these procedures, intraoperative biopsies of penile skin were obtained from areas with abnormal or suspicious tissue changes, as determined by the operating surgeon, reflecting routine clinical practice. Histopathological analysis of these samples demonstrated findings consistent with LS.

A total of 11 pediatric patients met the inclusion criteria for this study. Patient age ranged from 2 to 14 years, with a mean age of 6.1 years at the time of surgery. In total, 13 skin specimens were analyzed. Two patients required a second surgical intervention due to postoperative complications, and an additional biopsy sample was obtained during the revision procedure in each case.

Hypospadias severity was classified according to the anatomical location of the urethral meatus. Six patients (54.5%) presented with anterior hypospadias, three (27.3%) with midshaft hypospadias, and two (18.2%) with posterior hypospadias. Surgical technique was selected based on anatomical characteristics and intraoperative assessment.

Five patients underwent urethral reconstruction using the Mathieu technique, and two were treated using the meatal advancement and glanuloplasty incorporated (MAGPI) procedure. In three cases, urethroplasty was performed using an island flap technique. One patient required a staged repair utilizing the Byars technique due to the severity of the defect. In two patients who developed urethrocutaneous fistulas, surgical repair was performed using simple fistula closure in two layers.

All biopsy specimens were submitted for histopathological evaluation. Tissue samples were examined using conventional optical microscopy to identify characteristic features of LS, including epithelial atrophy, dermal hyalinization, and chronic inflammatory infiltration. The presence of koilocytic epithelial changes suggestive of HPV infection was also assessed.

Polymerase chain reaction (PCR) analysis was performed on all specimens for HPV detection and genotyping, including both low-risk and high-risk subtypes. The presence of viral DNA and specific serotypes were recorded and correlated with histopathological findings and clinical outcomes.

Postoperative follow-up data were obtained through review of clinical records. The mean follow-up period was 28.3 months (range 3-48 months). Postoperative complications, including urethrocutaneous fistula, meatal stenosis, urethral stenosis, and wound dehiscence, were recorded.

Continuous variables were summarized as means and ranges. Categorical variables were analyzed using Fisher’s exact test or the chi-square test, as appropriate. Student’s t-test was used for comparisons involving continuous variables. Statistical significance was defined as a p-value <0.05.

## Results

The clinical, histopathological, and virological characteristics of the patients are summarized in Table 1.

**Table 1:** Clinical, histopathological, and virological findings in patients undergoing hypospadias repair. Values are presented as number (percentage) unless otherwise indicated. HPV detection was performed using PCR with viral genotyping. LS: lichen sclerosus; HPV: human papillomavirus; PCR: polymerase chain reaction.

Variable	Value
Number of patients	11
Mean age	6.1 years
Skin specimens analyzed	13
Anterior hypospadias	6 (54.5%)
Midshaft hypospadias	3 (27.3%)
Posterior hypospadias	2 (18.2%)
LS histology	13 (100%)
Koilocytic changes	10 (76.9%)
HPV detected by PCR	6 (46.1%)
Overall complications	6 (54.5%)

The overall complication rate was 54.5%. The most frequent complications were meatal stenosis (n=3) and urethrocutaneous fistula (n=2), followed by urethral stenosis (n=1) and wound dehiscence (n=1). One patient presented with more than one complication.

Koilocytic changes were identified in 76.9% of specimens, and HPV DNA was detected in 46.1%. Among HPV-positive samples, genotypes 6, 16, 18, and 33 were identified. Two specimens obtained from the same patient during separate procedures tested positive for HPV.

No statistically significant association was found between HPV detection or koilocytic changes and postoperative complications (p>0.05).

The mean complication-free interval was 10.6 months (range 2-21 months). Management of complications included repeat urethroplasty with dartos flap interposition (n=2), urethral dilation (n=1), and staged urethral reconstruction (n=1). Two patients were lost to follow-up before definitive management.

## Discussion

LS has increasingly been recognized as a relevant condition in pediatric urology. Although traditionally considered uncommon in children, several reports have demonstrated that LS may occur in pediatric patients and may contribute to urethral pathology and complications following reconstructive surgery [1-3]. The chronic inflammatory nature of LS leads to progressive epithelial atrophy and dermal fibrosis, which can compromise the elasticity and integrity of genital tissues.

Hypospadias repair remains one of the most common reconstructive procedures in pediatric urology. Despite advances in surgical techniques, postoperative complications such as fistula formation, meatal stenosis, urethral stricture, and wound dehiscence remain relatively frequent. Reported complication rates vary depending on the severity of the defect, the surgical technique used, and surgeon experience [9,10]. In addition to technical and anatomical factors, the biological characteristics of the tissues used for urethral reconstruction may also influence surgical outcomes.

Inflammatory dermatoses such as LS may affect penile skin and the urethral plate, leading to chronic fibrosis and scarring. These pathological changes may compromise the long-term integrity of the reconstructed urethra and may contribute to complications such as meatal stenosis or urethral strictures [7,8]. In this context, LS has been increasingly recognized as a potential factor in failed or complicated hypospadias repairs.

Previous studies have reported histological evidence of LS in patients undergoing revision surgery for failed hypospadias repair. In such cases, inflammatory and fibrotic changes may extend into the urethral plate and adjacent tissues, potentially impairing wound healing and increasing the risk of postoperative complications [11]. For this reason, some authors recommend histological evaluation of suspicious penile tissue during reconstructive procedures, particularly in patients undergoing redo surgery.

The presence of LS may also influence surgical strategy. When LS involvement is suspected, the use of genital skin for urethral reconstruction may be suboptimal. Contemporary reconstructive techniques increasingly favor the use of extragenital grafts, such as buccal mucosa, particularly in complex or redo cases. Buccal mucosa provides a well-vascularized epithelial surface and is less likely to be affected by inflammatory dermatoses such as LS, which may improve long-term outcomes in selected patients [12,13].

Another aspect of interest in the pathogenesis of LS is its possible association with HPV infection. Koilocytic changes observed in epithelial cells are considered histological features suggestive of HPV infection. Previous investigations have identified HPV DNA in a subset of LS lesions, suggesting a potential role in epithelial injury or chronic inflammation [4,5]. However, the exact role of HPV in the pathogenesis of LS remains controversial.

In the present series, HPV DNA was detected in a substantial proportion of specimens, including both low-risk and high-risk viral subtypes. Despite this finding, no statistically significant association was identified between HPV detection and postoperative complications. These results suggest that HPV may be present in LS lesions without necessarily playing a direct role in the development of surgical complications.

Another important consideration in hypospadias surgery is the timing of complication development. Several studies have shown that most postoperative complications become clinically evident during the first postoperative year, although late complications may still occur. Consequently, long-term follow-up is essential for accurately assessing surgical outcomes and identifying delayed complications [10,14-16].

The present study has several limitations. The relatively small sample size limits statistical robustness and generalizability. The retrospective design precludes establishing causal relationships between histopathological findings and surgical outcomes. In addition, the intraoperative sampling of tissue based on abnormal or suspicious appearance may introduce selection bias, as normal-appearing tissue was not systematically evaluated. This approach reflects routine clinical practice but may limit the ability to determine the true prevalence of LS and HPV-related changes. Furthermore, although molecular testing for HPV was performed, the interpretation of its clinical significance remains limited by the sample size.

Despite these limitations, the findings of this study suggest that LS may represent an underlying pathological factor in a subset of patients with complications after hypospadias repair. Increased awareness of this condition may help surgeons better evaluate the tissues used for urethral reconstruction and consider alternative surgical strategies when LS involvement is suspected. These findings support careful assessment of penile tissue during hypospadias reconstruction and suggest that histopathological evaluation may be useful in selected cases, particularly in patients undergoing revision surgery.

Further multicenter studies including larger patient populations and standardized histopathological and molecular evaluation are needed to better clarify the role of LS in hypospadias surgery and to determine the clinical significance of HPV detection in these lesions.

## Conclusions

LS was consistently present in skin samples obtained during hypospadias repair in this series and may represent an important pathological factor associated with postoperative complications. HPV was detected in a subset of cases; however, its role in surgical outcomes remains unclear. Further studies with larger patient populations are needed to better understand the relationship between LS, HPV infection, and reconstructive outcomes in pediatric hypospadias surgery.
